# Sengstaken–Blakemore Tube Placement: A Simulation-Based Training Program for a High-Acuity, Low-Frequency Procedure

**DOI:** 10.15766/mep_2374-8265.11613

**Published:** 2026-06-24

**Authors:** Carolyn Wilson, Ceena Chandrabos, Katie Shen, Manu Venkat, Deepak Pradhan, Adam Goodman, Renee Williams, Catherine Uy

**Affiliations:** 1 Resident, Department of Medicine, New York University Langone Health; 2 Assistant Professor, Division of Gastroenterology and Hepatology, Department of Medicine, New York University Langone Health; 3 Fellow, Division of Gastroenterology and Hepatology, Department of Medicine, New York University Langone Health; 4 Associate Professor, Division of Pulmonary, Critical Care, and Sleep Medicine, Department of Medicine, New York University Langone Health; 5 Section Chief, Division of Gastroenterology and Hepatology, Department of Medicine, New York University Langone Health; 6 Associate Professor, Division of Gastroenterology and Hepatology, Department of Medicine, New York University Langone Health; 7 Clinical Instructor, Division of Gastroenterology and Hepatology, Department of Medicine, New York University Langone Health

**Keywords:** Blakemore, Simulation, Esophageal Varices, Competency-Based Medical Education, Quality Improvement/Patient Safety, Critical Care Medicine, Gastroenterology, Clinical/Procedural Skills Training

## Abstract

**Introduction:**

Variceal hemorrhage is a severe and often fatal consequence of portal hypertension from liver cirrhosis. Although endoscopic and radiographic interventions have largely replaced balloon tamponade, the Sengstaken–Blakemore tube (SBT) remains a critical salvage therapy. However, declining use has led to limited provider experience and familiarity with SBT placement and management.

**Methods:**

We implemented a hands-on, manikin-based simulation program for fellows and attendings from gastroenterology and critical care to improve provider familiarity with SBT placement and management. Standardized SBT kits were developed and distributed across all sites. Annual simulation sessions included presession didactics, live demonstrations, and small-group hands-on practice. Pre- and postsession surveys assessed self-reported confidence in SBT placement, management, and removal using a 5-point Likert scale. A Wilcoxon signed-rank test analyzed changes in confidence.

**Results:**

Fifty-four participants participated in 2023. Prior to training, only 20% of participants reported having received any formal instruction on SBT placement or management and 39% had never placed an SBT in a real-life scenario. Confidence in SBT placement (mean 3.00-4.30), management (1.78-4.31), and removal (2.17-4.43) significantly increased after the simulation.

**Discussion:**

This simulation-based intervention was associated with improved self-reported confidence in SBT placement, management, and removal for a high-acuity, low-frequency procedure. Given interest, emergency medicine, surgery, and internal medicine were included in subsequent years. Our results underscore the importance of structured training in bridging knowledge and skill gaps across gastroenterology and critical care disciplines. Future work should assess long-term retention and explore curriculum integration.

## Educational Objectives

By the end of this simulation-based training, learners will be able to:

1. Identify appropriate clinical indications, contraindications, and potential complications associated with Sengstaken–Blakemore tube (SBT) placement in the setting of refractory variceal hemorrhage.
2. Demonstrate the key steps in the preparation, deployment, and securement of an SBT using manikin-based simulation.
3. Outline essential components of postplacement management, including monitoring protocols, risk mitigation, and appropriate communication with multidisciplinary teams.
4. Reflect on self-reported confidence in performing and teaching SBT placement through pre- and posttraining self-assessment.

## Introduction

Variceal hemorrhage (VH) is a severe and often fatal consequence of portal hypertension, most commonly arising in the setting of cirrhosis.^1^ Mortality associated with esophageal variceal bleeding remains substantial, with 5-year mortality approaching 38%.^2^ Guidance from Baveno VII recommends nonselective beta blockade or screening/surveillance endoscopies as a preventative strategy to mitigate portal hypertensive complications, such as VH.^3^ Historically, supportive transfusions, pharmacological therapies, and balloon tamponade were the primary modalities of addressing acute variceal bleeding. Specifically, the Sengstaken–Blakemore tube (SBT) allows for balloon tamponade when correctly positioned with the gastric balloon anchored in the fundus, thus temporarily reducing the portal pressure in the esophagus and achieving hemostasis in patients with suspected or confirmed gastroesophageal varices. This has largely been replaced by endoscopic esophageal band ligation (EBL) and sclerotherapy using agents such as sodium tetradecyl sulfate. However, in situations where EBL or sclerotherapy is unavailable or unsuccessful, temporizing measures are needed to bridge the patient to more definitive therapies such as transjugular intrahepatic portosystemic shunt (TIPS) and/or liver transplant.^4^ These salvage therapies include balloon tamponade, as well as covered self-expanding esophageal stents (SEMS), which is limited by product availability and limited expertise by general gastroenterologists.^1^

The clinical use of SBTs has declined due to improved access to endoscopic and radiologic interventions, alongside better prophylactic management of portal hypertension.^4^ While providing a lifesaving therapy, SBT utilization carries significant risks, including esophageal perforation, mucosal ischemia, aspiration, and airway obstruction due to tracheal compression. Current guidelines endorse SBT utilization only in emergent settings where other modalities are either ineffective or unavailable.^5^ Despite these risks, outcomes remain meaningful; in 1 cohort, 59% of patients survived to hospital discharge and 41% were alive at 1 year.^6^ However, decreased utilization and limited dedicated training has led to a lack of preparedness in SBT deployment in emergent settings. A study by Rutter et al.^7^ showed that only 38% of gastroenterology (GI) trainees felt confident to insert an SBT independently and 25% felt confident to give management instructions to nursing or junior medical staff, whereas 100% of trainees felt there should be formal training. In a recent study,^8^ both GI fellows and faculty demonstrated low baseline knowledge and procedural skill with SBT placement, with no significant difference between the 2 groups prior to simulation-based training. After a targeted simulation intervention, both groups showed significant improvement in knowledge and skill, and faculty reported increased confidence in teaching the procedure.^8^ These findings highlight that the knowledge and skill gap affects both trainees and faculty, likely due to the infrequent clinical use of SBT and the lack of standardized curricula. Inadequate preparation for SBT deployment may compromise patient outcomes and lead to undue complications.^9^ These findings underscore the need for formal training for SBT placement as a high-acuity, low-frequency procedure and support future work using more robust assessment methods.

In the academic year of 2021–22, there were at least 5 instances within our academic center where SBT placement was warranted. During these instances, difficulties were encountered resulting in delays in patient care as well as 1 adverse event. There were several barriers to successful deployment of the SBT, including obtaining materials, lack of procedural familiarity, and limited knowledge of management after placement. Our project aimed to create comprehensive and easily accessible SBT kits, conduct manikin-based simulation training, review the steps of SBT placement and postplacement management, and assess change in participants’ self-reported confidence before and after the training sessions. Simulation was chosen as the desired modality because it provides a controlled environment for practicing psychomotor and crisis management skills without risk to patients, directly supporting patient safety goals. Guided by Kolb's experiential learning cycle^10^ and Ericsson's deliberate practice model,^11^ the curriculum incorporated hands-on simulation, stepwise task deconstruction, and iterative practice with structured feedback. Aligned with Miller's Pyramid, simulation advances learners from knowledge to performance, bridging “knows how” to “shows how” and preparing them for clinical application. We embedded systems-focused objectives, including standardizing procedural steps, optimizing team roles, and identifying latent safety threats during debriefings, and outlined follow-up measures, including evaluating learner confidence, monitoring clinical performance indicators, and refining the curriculum based on identified gaps.

## Methods

### Development

We worked with key stakeholders, which included GI faculty, fellows, and leadership from our simulation center at a quaternary academic health system who had prior experience with Blakemore placement across our hospitals. We identified 3 main areas of improvement: the need for a comprehensive Blakemore kit, training sessions for faculty and trainees from both GI and critical care, and to assess participants’ level of confidence with performing this procedure after our training session. The case was created as a procedure embedded within a larger simulation case, which would allow trainees to consider assessment and stabilization of a critically ill patient in addition to task training. The total simulation session took around 90 minutes.

### Equipment

The following materials are required to conduct the simulation case on SBT placement:

• SBT comprehensive kits (Appendix A)
• Standard adult-size airway task trainer or high-fidelity manikin with an oropharyngeal passage that can accommodate an SBT; the manikin should be positioned supine with the head elevated to 30 degrees
• IV pole

### Personnel

In each simulation, learners were GI fellows and attendings and critical care attendings. The simulations were facilitated by GI attendings and fellows with prior knowledge of SBT placement, with 1 to 2 facilitators per station. The leads who were GI attendings included advanced gastroenterologists and hepatologists who volunteered to do so, and GI fellows who had real-world experience with SBT placement. Subsequent GI fellows who led the sessions were those who had attended at least 1 training session and had demonstrated proper SBT placement technique with 1 of the original instructors. The training was implemented in August, as this was the beginning of the academic year. First-year GI fellows, transplant hepatology fellows, and newly hired GI attendings were prioritized for the training. Staff from the simulation center were also available for any technical support during the simulation training. The group was debriefed as a whole with the team that designed the simulation.

### Implementation

We developed standardized Blakemore kits (see Appendix A) and placed them in endoscopy suites at all 4 hospital sites affiliated with our GI fellowship. We ensured that the same kits were used during the simulation training to ensure familiarity. In settings where commercially available premade SBT kits are available, training sessions should utilize the same kits.

Simulation sessions (Appendix B) were open to new trainees and returning providers seeking to reinforce procedural skills. Initially, the session targeted trainees and physicians within the GI division. Providers from the pulmonary and critical care division expressed interest in participating and were included in the session's initial iteration as well. The sessions were held in July and August, a time chosen to align with the start of the academic year and maximize utility for incoming GI fellows. This program was initially offered once yearly to align with the academic calendar and existing institutional procedural training structures, although the optimal retraining interval remains uncertain. Prior to the session, participants received required preparatory materials, including a step-by-step SBT placement checklist (Appendix C) and an optional instructional video.^12^ The instructional video was made optional to accommodate varying learner schedules, allow presession review, and avoid redundancy during the live in-session demonstration while still serving as an accessible resource before and after the training. Participants also completed a pretraining survey (Appendix D).

Hands-on sessions were 90 minutes long and held at NY Simulation Center, a large urban, academic simulation center dedicated to health professions education. Half of the room was arranged as a seminar space for the introductory learning, and the other half contained 4 simulation stations, each with an SBT kit and simulation manikin. To maximize hands-on experience, each simulation station was limited to 3–4 participants with its own dedicated instructor. This resulted in an enrollment limit of 12-16 participants per session.

Each session's prebrief began with facilitator and participant introductions, followed by a group didactic session in slideshow format reviewing indications, contraindications, and equipment logistics. This was followed by a group demonstration of the full SBT placement process. Learners were then divided into small groups of 3-4 and assigned to manikin stations led by an instructor. Each participant practiced SBT placement on the manikin, receiving instructor-led direct feedback in real time. The step-by-step checklist in Appendix C was used as a formative teaching guide during deliberate practice to reinforce correct sequencing and highlight key safety steps in placement, management, and removal, such as confirming that participants remove the counterweight first, reassess hemodynamics and blood reflux, and sequentially deflate the gastric and then esophageal balloons. If a learner omitted, partially completed, or incorrectly performed any component of the procedure, the instructor provided immediate corrective feedback, and the learner was required to repeat the procedure from the beginning until all steps were performed correctly. Formal checklist scores were not collected or analyzed as study outcomes. Participants completed a posttraining survey (Appendix E) at the end of the session. Instruction was 20 minutes, hands-on practice 20 minutes, assessment 10 minutes, and debriefing 20 minutes.

### Debriefing

Following the manikin-based simulations, a structured debriefing was conducted using the Gather-Analyze-Summarize (GAS) framework to review postplacement management and care instructions. As our program spans multiple hospitals, attention was paid in this section to site-specific logistical details such as kit locations and hospital policies for postplacement management. Upon ending the session, participants were reminded to save the presession emails containing SBT placement instructions and the optional YouTube video to use as resources if they were called upon to place an SBT in clinical practice.

### Assessment

Surveys were used to gather participant demographics, baseline experience with SBT placement and management, and self-reported confidence levels before (see Appendix D) and after (see Appendix E) training using a 5-point Likert scale. These surveys were distributed electronically immediately prior to instruction and during the debrief session after the hands-on workshop. The survey items were reviewed for clarity prior to implementation; however, the response scale did not include behavioral anchors linked to specific observable competencies, a limitation addressed in the Discussion section. The survey included statements such as:

• How confident were you with Blakemore tube placement BEFORE/AFTER training?
• How confident were you with Blakemore management BEFORE/AFTER training?
• How helpful was the SIM session in learning/facilitating Blakemore placement?

For each item, participants rated their comfort level using a 5-point Likert scale (1 = *very uncomfortable*, 2 = *uncomfortable*, 3 = *neutral*, 4 = *comfortable*, 5 = *very comfortable*). Both pre- and postsimulation surveys were administered anonymously immediately before and after the session. Within the New World Kirkpatrick model, these outcome measures primarily reflect learner reaction and self-perceived learning following training. Descriptive statistics were performed, and a Wilcoxon signed-rank test was conducted to determine if there was significant difference in confidence before and after the workshop using IBM SPSS v25.^13^

## Results

**Figure 1.**
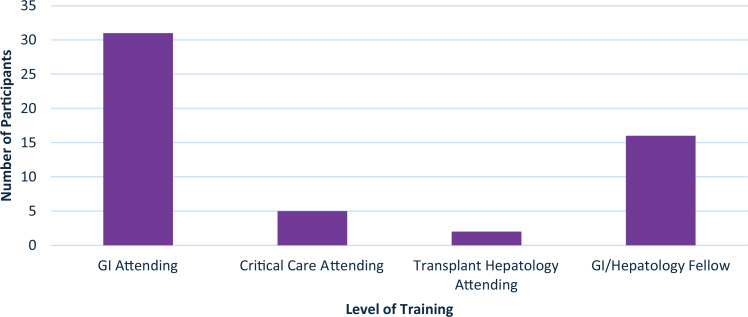
Level of training among participants in the SBT simulation program. Abbreviations: SBT, Sengstaken–Blakemore tube, GI, gastroenterology.

**Figure 2.**
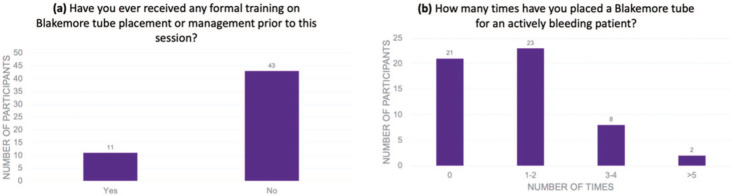
(A) Proportion of simulation participants with prior formal training in SBT placement. (B) Self-reported frequency of prior SBT placement among simulation participants. Abbreviation: SBT, Sengstaken–Blakemore tube.

A total of 54 participants participated in 2023 in a hands-on simulation, which included 31 GI attendings, 16 GI fellows, and 5 critical care attendings (Figure 1), with a 100% response rate. Prior to the training, only 20% of participants reported having received any formal instruction on SBT placement or management (Figure 2A). A substantial proportion (39%) had never placed an SBT in a real-life scenario, whereas 43% had placed an SBT between 1 and 2 times, 15% had done so 4 to 5 times, and just 4% had performed 5 or more placements (see Figure 2B).

Participants demonstrated significant improvement in self-reported confidence after, compared with before, training across all measured domains (Table). For SBT placement, only 9% of participants reported being somewhat or very confident prior to the training. This increased dramatically to 91% following the session. The mean confidence score rose from 3.00 (*SD* = 1.97) pretraining to 4.30 (*SD* = 0.63) posttraining on a 5-point scale. Similarly, for SBT postplacement management, 7% of participants reported being somewhat or very confident prior to training, compared to 91% afterward. The mean confidence score increased from 1.78 (*SD* = 1.08) to 4.31 (*SD* = 0.63). Confidence in SBT removal also improved notably. Prior to training, only 20% of participants were somewhat or very confident, whereas 94% felt confident following the session. The mean confidence score increased from 2.17 (*SD* = 1.33) to 4.43 (*SD* = 0.60).

**Table.**
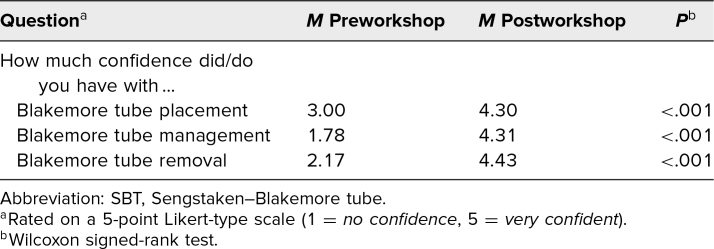
Pre/Postworkshop Confidence in SBT Placement, Management, and Removal (*N* = 54)

In terms of training effectiveness, participants rated the hands-on simulation component most favorably. The hands-on session was deemed very helpful by 96% of respondents, with a mean rating of 4.96 (*SD* = 0.19). The instructional checklist was rated as very helpful by 63% of participants, yielding a mean score of 4.59 (*SD* = 0.56). The video tutorial was considered very helpful by 54%, with a mean rating of 4.31 (*SD* = 1.05).

Open-ended feedback further highlighted the perceived value of the training. Many participants expressed a desire for the course to be offered annually or biannually and requested supplemental materials such as printed handouts, dot phrases to aid in documentation, and postplacement care instructions. Suggestions for improvement included incorporating common pitfalls, such as avoiding esophageal perforation, and integrating clinical case examples. Overall, the session was widely regarded as effective, well organized, and essential for building procedural self-confidence for high-acuity, low-frequency interventions like SBT placement. Participants also noted that the Blakemore instruction sheet was helpful for remembering the key steps for placement, and the kits consolidated all needed equipment. Additional study is needed to assess the impact on patient safety outcomes.

## Discussion

Our experience at a large academic center reinforces that SBT placement is a high-risk, low-frequency procedure for which clinicians may have limited training and inconsistent access to accessory supplies needed for safe deployment. SBT placement carries important risks stemming from both insertion and balloon inflation. Minor complications include nasal or pharyngeal mucosal irritation, epistaxis with nasal insertion, hoarseness, and nasal bridge pressure ulcers from prolonged tension. Major complications arise largely from malposition or excessive balloon pressure and may include esophageal rupture (reported in up to 6% of cases), esophageal necrosis, aspiration pneumonia when the airway is unprotected, balloon migration leading to loss of tamponade, and airway compromise. Catastrophic events, although rare, may involve massive hemorrhage due to balloon dislodgement or fistula formation, tracheal compression causing complete airway obstruction, and cardiopulmonary arrest from hypoxia or exsanguination. Together, these reflect the high-risk nature of the procedure and the need for rigorous training and vigilance during placement and monitoring.^4^

The survey indicated that despite being from a high-volume academic institution, the majority of participants, including both trainees and experienced attendings, had limited or no prior formal education regarding SBT placement or management, which correlated to their minimal confidence in this procedure. Our initiative demonstrates that a structured, hands-on simulation session significantly improves provider confidence in the use of SBTs among GI and critical care physicians.

Following participation in a targeted simulation session, participants reported significantly greater confidence with SBT placement, postplacement management, and removal. These findings suggest that even brief, focused simulation-based training may improve perceived familiarity with a rarely performed, high-stakes procedure. Importantly, this training included both GI fellows and attendings and critical care attendings, reflecting the interdisciplinary nature of acute gastrointestinal bleeding management and the need for cross-specialty procedural readiness. Emergency medicine and internal medicine participants were not included in the initial cohort because the curriculum was first piloted with smaller GI and critical care groups to allow iterative refinement of the curriculum; however, these specialties were incorporated in subsequent cohorts to increase generalizability.

Our findings align with growing literature supporting the value of simulation-based education for infrequent but high-stakes clinical scenarios. In this context, greater clinician familiarity and self-reported confidence with SBT use may be valuable, particularly when balloon tamponade must be deployed rapidly as a temporizing intervention to avoid the high morbidity and mortality associated with uncontrolled variceal bleeding. However, because we did not collect objective performance or clinical outcome data, the present findings should be interpreted as evidence of feasibility, acceptability, and improved self-reported confidence rather than verified procedural competence.

Challenges faced during the implementation process included obtaining enough task trainers for our simulations, creating simulation kits, and ensuring that all 4 hospital sites remained stocked with SBT kits. To have enough task trainers, we borrowed additional from the emergency medicine department. We used expired medical supplies to create our simulation kits. To ensure that Blakemore kits were stocked, we created a document detailing the necessary components of a Blakemore kit with pictures of the final assembled package, and communicated with the endoscopy charge nurses at all 4 sites to ensure that each site had 2 complete kits. Further data is needed to assess if there was increased incidence in SBT placement after trainings were completed.

Despite the strengths of our initiative, including interdisciplinary participation and pre/post assessment, several limitations should be noted. First, we did not assess long-term retention of procedural knowledge or self-reported confidence. Second, we did not collect formal objective performance data, such as checklist scores or knowledge-based testing, and therefore cannot conclude that technical skill or clinical decision-making improved because of the training. The checklist in Appendix C was used for formative instruction and feedback during the session rather than as a scored study outcome. Third, our assessment relied on self-reported confidence measures, which reflect learner perceptions rather than objectively verified procedural competence. Fourth, the surveys used a 5-point Likert scale without behavioral anchors tied to defined competency benchmarks; this may have introduced variability in how learners interpreted the scale and increased subjectivity. Finally, simulation cannot fully replicate the complexity of real-world SBT placement, particularly in anatomically challenging or hemodynamically unstable patients, which may limit transferability to clinical practice.

Potential future directions include exploring a formal certification process for Blakemore training and assessing for improvement in clinical outcomes or reductions in procedure-related complications after the implementation of our simulation sessions. As a future direction, we aim to analyze outcomes from our expanded learner cohort—including emergency medicine, internal medicine, and surgery—to determine how the curriculum performs across a wider range of clinical disciplines. Our study demonstrated high learner satisfaction and significant improvement in self-assessed confidence. Our simulation had significant clinical impact, as the SBT kits are now included in the GI emergency travel carts and available across all our sites and has been incorporated into the variceal bleeder workflow. This is consistent with level 3 (behavior change in clinical practice) of the Kirkpatrick model. Although we did not assess level 4 (impact on patient outcomes) of the Kirkpatrick model, these will be important areas for future study. Even though the program was initially implemented annually to align with existing institutional procedural training structures, more frequent refresher sessions may be warranted for this low-frequency procedure. Future studies should therefore examine longitudinal skill retention and determine the optimal retraining interval, integration of simulation into routine training for gastrointestinal emergencies, and assess its impact on patient care metrics.

## Appendices


Components of SBT Kit.docxSimulation Case.docxBlakemore Tube Placement Checklist.docxBlakemore Placement Pretraining Survey.docxBlakemore Placement Posttraining Survey.docx
*All appendices are peer reviewed as integral parts of the Original Publication.*
