# Supplementary material for: Sengstaken–Blakemore Tube Placement: A Simulation-Based Training Program for a High-Acuity, Low-Frequency Procedure
Source: MedEdPORTAL. 2026 Jun 24;22:11613. doi: 10.15766/mep_2374-8265.11613 (PMC13291162; doi:10.15766/mep_2374-8265.11613)
Supplement: Supplementary file 1 — Components of SBT Kit.docxSimulation Case.docxBlakemore Tube Placement Checklist.docxBlakemore Placement Pretraining Survey.docxBlakemore Placement Posttraining Survey.docx [file mep_2374-8265.11613-s001.zip › B. Simulation Case.docx]

| Appendix B: Simulation Case  SIMULATION CASE TITLE: Emergency Management of Variceal Hemorrhage Requiring Sengstaken–Blakemore Tube Placement  AUTHORS: Carolyn Wilson MD, Katie Shen MD, Ceena Chandrabos MD, Catherine Uy MD, Manu Venkat MD, Deepak Pradhan, MD, Adam Goodman MD, Renee Williams, MD  LEARNER AUDIENCE: Gastroenterology, internal medicine, critical care, emergency medicine, and surgery fellows/faculty | |
| --- | --- |
| PATIENT NAME: John Smith  PATIENT AGE: 62  CHIEF COMPLAINT: Vomiting blood  PHYSICAL SETTING: Emergency Department | |
| Brief Narrative Description of Case | *A 62-year-old male with known alcohol-associated cirrhosis presents with hematemesis, hypotension, and tachycardia. Learners must recognize the indication for and demonstrate appropriate placement of a Sengstaken–Blakemore tube, manage the resuscitation, and anticipate complications. The goal is to prepare learners for this high-acuity, low-frequency procedure in a safe, simulated setting.* |
| Primary Learning Objectives | *By the end of this simulation-based training, learners will be able to:*   1. *Identify appropriate clinical indications, contraindications, and potential complications associated with Sengstaken–Blakemore tube (SBT) placement in the setting of refractory variceal hemorrhage.* 2. *Demonstrate step-by-step competency in the preparation, deployment, and securement of a SBT using manikin-based simulation.* 3. *Outline essential components of post-placement management, including monitoring protocols, risk mitigation, and appropriate communication with multidisciplinary teams.* 4. *Reflect on personal confidence and readiness to perform and teach SBT placement through pre- and post-training self-assessment.* |
| Critical Actions | 1. *Recognize ongoing variceal hemorrhage despite standard medical therapy.* 2. *Ensure airway protection via endotracheal intubation prior to SBT placement.* 3. *Correctly assemble all necessary equipment (SBT, syringes, manometer, suction, etc.).* 4. *Test balloon integrity and flush lumens prior to insertion.* 5. *Insert SBT to proper depth (~50 cm), inflate gastric balloon, confirm placement via chest/abdominal Xray or endoscopy.* 6. *Secure tube and, if needed, inflate esophageal balloon while monitoring pressure.* 7. *Identify potential complications of placement.* 8. *Initiate transfer to ICU and coordinate with GI/surgery for definitive management.* |
| Learner Preparation or Prework | *Prior to the session, participants should review required preparatory materials, including a step-by-step SBT placement checklist and an optional instructional YouTube video.*  [*https://www.youtube.com/watch?v=brkB6TTeOqk*](https://www.youtube.com/watch?v=brkB6TTeOqk) |

| Initial Presentation | | | |
| --- | --- | --- | --- |
| Initial Vital Signs | HR: 122 bpm  BP: 84/46 mmHg  RR: 22  Temp: 36.8°C  SpO₂: 95% on non-rebreather mask | | |
| Overall Setting and Appearance | *Learners enter a high-acuity emergency department resuscitation bay. The mannequin is an adult male lying supine, intubated or in respiratory distress, with simulated blood in the mouth and emesis basin. Suction, crash cart, and IV poles are visible. Nurses appear distressed and call for immediate assistance due to continued hematemesis despite interventions.* | | |
| Standardized Participants (and Their Roles in the Room at Case Start) | *Nurse (played by facilitator): “He’s still vomiting blood. GI is tied up in another emergency. We gave octreotide and antibiotics, but he's not stabilizing.”*  *Respiratory Therapist (optional): Assists with airway management. Can confirm if patient is already intubated or needs to be intubated based on learner decision.* | | |
| HPI | *62-year-old man with cirrhosis presents with hematemesis for the past 3 hours.*  *Worsening lethargy and dizziness.*  *Has had 2 prior admissions for variceal bleeding with banding, on most recent admission patient refused TIPS.*  *Denies NSAID or alcohol use in past week.*  *Vomited “a large amount” of blood twice en route to hospital.*  *Learners must ask about cirrhosis history and previous GI bleeds.* | | |
| Past Medical/Surgical History | Medications | Allergies | Family History |
| Alcohol-associated cirrhosis, portal hypertension, esophageal varices with prior banding  No prior surgeries | Carvedilol  Furosemide  Spironolactone | NKDA | Non-contributory |
| Physical Examination | | | |
| General | Appears pale, diaphoretic, altered mental status, ongoing hematemesis | | |
| HEENT | Pale mucosa, blood in oropharynx | | |
| Neck | No JVD, trachea midline | | |
| Lungs | Coarse breath sounds | | |
| Cardiovascular | Tachycardic, peripheral pulses weak, cap refill >3s | | |
| Abdomen | Distended, positive fluid wave, mild RUQ tenderness, no rebound/guarding | | |
| Neurological | Drowsy but arousable to verbal stimuli | | |
| Skin | Cool, clammy, no jaundice | | |
| GU | Foley in place, low urine output | | |
| Psychiatric | Unable to assess due to clinical condition | | |

| Instructor Notes - Changes and CASE Branch Points | | |
| --- | --- | --- |
| Intervention / Time Point | Change in Case | Additional Information |
| 5 minutes into the case with no intervention | BP drops to 80/40, HR increases to 130 | RN prompts: “Doctor, the bleeding is getting worse! His pressure just dropped again.” |
| Learners initiate fluid resuscitation and blood transfusion | BP stabilizes to 95/60, HR down to 110 | RN says: “Vitals are improving, but he’s still bleeding heavily.” |
| Octreotide 50mcg bolus followed by 50mcg/hr rate and third generation cephalosporin IV administered | Bleeding continues but progression slows | No improvement in mental status; patient remains altered |
| Learners decide to intubate | Oxygenation improves, aspiration risk mitigated | RT confirms: “Tube placement verified. Breath sounds bilaterally present.” |
| Ongoing bleeding but GI unavailable | Bleeding ongoing, BP again drops to 80/50, HR to 130s | RN says: “He’s bleeding more and GI is saying they’re scoping another variceal bleed right now.” |
| SBT equipment is gathered and gastric balloon is inflated initially to 50cc, SBT placement confirmed with CXR, and gastric balloon then inflated fully to 250cc | Bleeding temporarily controlled, HR decreases to 100, BP to 100/65 | RN: “The bleeding seems to have slowed since the balloon went in.” |
| Esophageal balloon is inflated without pressure monitoring | Patient becomes bradycardic, hypotensive, possible esophageal rupture | RN: “His vitals are crashing!” Recognizes risk of esophageal rupture if balloon pressure is not checked |
| Proper traction applied with saline bag, second CXR taken to ensure gastric balloon is fully inflated, and importance of marking exact location of SBT at ETT is emphasized to minimize risk of slippage | Patient stabilizes with BP 110/70, HR 95 | Learners are prompted to consider next steps (GI/IR evaluation, ICU transfer, documentation of balloon inflation time, location of SBT at ETT) |

Ideal Scenario Flow

Learners enter the resuscitation bay to find a patient with cirrhosis experiencing ongoing hematemesis and hypotension. The patient is diaphoretic, pale, and lethargic. Learners quickly place the patient on monitors and recognize the need for aggressive resuscitation. They administer IV fluids, and start octreotide and antibiotics.

After assessing airway risk, the team performs endotracheal intubation to protect against aspiration. Despite initial interventions, the patient remains unstable and continues to have hematemesis. Learners recognize the need for balloon tamponade and gather the equipment for Sengstaken–Blakemore tube placement.

The team checks balloon integrity, prepares suction, and inserts the SBT to 50 cm. They inflate the gastric balloon to 50cc, confirm position via imaging, inflate the gastric balloon to 250cc, get repeat imaging to make sure the balloon is fully inflated, and apply traction. It is good practice to inflate the gastric balloon with Omnipaque or other readily-available radio-opaque solution that can be easily visualized on bedside CXR. Bleeding slows. Location of SBT at ETT is marked to minimize risk of slippage.

This stepwise approach is intended to confirm appropriate intragastric positioning and provide initial temporizing control of bleeding; it does not imply isolated gastric variceal hemorrhage. During the simulation, instructors emphasize that gastric balloon inflation is the standard first step for suspected esophageal variceal bleeding, as the gastric balloon anchors the device. Inflation of the esophageal balloon is reserved only for persistent bleeding despite adequate gastric balloon tamponade, serving as a key diagnostic and therapeutic decision point. If esophageal balloon inflation is indicated, learners monitor pressure carefully to avoid overinflation. Once stabilized, the team prepares for ICU transfer and communicates need for emergent GI intervention.

Anticipated Management Mistakes

1. Failure to protect the airway prior to SBT placement: Learners may attempt to insert the SBT without intubation, risking aspiration or inability to control the airway during active bleeding.
2. Improper balloon inflation technique or omission of pressure checks: Learners may inflate gastric without confirming placement, risking esophageal rupture. If esophageal balloon inflation is indicated, learners may not remember that a sphyngomanometer is needed to measure pressure. Reinforcement of safe inflation protocols is critical.
3. Delayed recognition of SBT indication: Learners may exhaust all medical therapies before considering balloon tamponade, delaying life-saving intervention. Case is designed to prompt early consideration in ongoing bleeding.
4. Failure to monitor and document balloon placement and timing: Learners may neglect marking SBT location and/or documentation of balloon pressure or inflation time, risking SBT migration and patient harm. Emphasize importance during debriefing, and how all providers including nursing and imaging technicians must be aware of this, as patients often need imaging after SBT placement as part of TIPS evaluation.
