# Supplementary material for: Sengstaken–Blakemore Tube Placement: A Simulation-Based Training Program for a High-Acuity, Low-Frequency Procedure
Source: MedEdPORTAL. 2026 Jun 24;22:11613. doi: 10.15766/mep_2374-8265.11613 (PMC13291162; doi:10.15766/mep_2374-8265.11613)
Supplement: Supplementary file 1 — Components of SBT Kit.docxSimulation Case.docxBlakemore Tube Placement Checklist.docxBlakemore Placement Pretraining Survey.docxBlakemore Placement Posttraining Survey.docx [file mep_2374-8265.11613-s001.zip › C. Blakemore Tube Placement Checklist.docx]

**Appendix C:** Blakemore Tube Placement Checklist

|  | **Blakemore Tube Placement** | Done correctly | Partially Done / Done Incorrectly | Not Done | N/A |
| --- | --- | --- | --- | --- | --- |
| 1 | Review indications (uncontrolled esophageal or gastric variceal hemorrhage refractory to pharmacologic and endoscopic therapy) and contraindications (unprotected airway, known esophageal rupture, recent esophageal surgery, caustic ingestion) for patient |  |  |  |  |
| 2 | Review relevant labs (CBC, INR/PTT, fibrinogen, CMP) and relevant imaging (recent CXR/CT if available) |  |  |  |  |
| 3 | Review Video (BAVLS) on Blakemore tube insertion as needed |  |  |  |  |
| 4 | Setup and preparation (assemble complete SBT kit; place patient in 30–45° head-of-bed elevation with neck midline; ensure suction ready; confirm team roles) |  |  |  |  |
| 5 | Place stopcock and adaptors into Blakemore tube |  |  |  |  |
| 6 | Test gastric and esophageal balloons in water to ensure there is no leak using syringe and 50cc of air. |  |  |  |  |
| 7 | Have portable X-ray at bedside (or ensure endoscopy team is present if performing endoscopic confirmation) |  |  |  |  |
| 8 | Use generous amount of lubricant on tube and place through the mouth to at least the 50cm mark |  |  |  |  |
| 9 | Either perform endoscopy to ensure proper balloon placement in the stomach or fill gastric balloon with 50cc of air |  |  |  |  |
| 10 | If endoscopy not performed, obtain chest/abd. X-ray and ensure gastric balloon is below diaphragm, in stomach. |  |  |  |  |
| 11 | Once gastric placement is confirmed with either endoscopy or x-ray, add an additional 200cc of air into the gastric balloon (total 250cc of air). |  |  |  |  |
| 12 | Place Kelly clamp on Blakemore tube gastric port to ensure air does not leak out |  |  |  |  |
| 13 | If Balloon inflation was not confirmed endoscopically, obtain another chest X-ray to ensure complete gastric balloon inflation has occurred. |  |  |  |  |
| 14 | Tie one end of Kerlex to the Blakemore tube, the other end to a 1L bag of IV fluid. |  |  |  |  |
| 15 | Pull Blakemore tube taught and place IV bag over an IV pole in order to provide continued pressure (typically 0.5–1 lb of traction) |  |  |  |  |
| 16 | If continued bleeding is noted, the esophageal balloon can then be inflated at the discretion of the treating physician. |  |  |  |  |
| 17 | The manometer should be attached to the esophageal port and inflated to a pressure of 48cm-61cm H2O (35mm-40mmHg – check manometer as this can vary) |  |  |  |  |
| 18 | Obtain another chest X-ray to ensure both balloons are inflated. |  |  |  |  |
| 19 | Consider using 2^nd^ Endotracheal tube fastener to secure balloon |  |  |  |  |
| 20 | If ET fastener used, remove Kerlex and IV fluid bag once fastened and mark tube with Sharpie pen at the ET connector to monitor for slippage. |  |  |  |  |
| 21 | Clean-up and restore bed/patient to pre-procedure conditions |  |  |  |  |
| 22 | Write EPIC procedure note (including volumes instilled, pressures used, traction weight, and confirmation method) |  |  |  |  |

|  | **Blakemore Tube Removal** | Done correctly | Partially Done / Done Incorrectly | Not Done | N/A |
| --- | --- | --- | --- | --- | --- |
| 1 | Ensure there is a plan for definitive hemostasis (confirmation of control via endoscopy or TIPS planning) |  |  |  |  |
| 2 | Unclamp the esophageal balloon port |  |  |  |  |
| 3 | Using a syringe, aspirate the air from the esophageal balloon port |  |  |  |  |
| 4 | Observe for signs of rebleeding for 4 hours |  |  |  |  |
| 5 | Remove the traction from the tube |  |  |  |  |
| 6 | Secure the tube to the nose of cheek using tape |  |  |  |  |
| 7 | Unclamp the gastric balloon port |  |  |  |  |
| 8 | Using a syringe, aspirate all the air from the gastric balloon |  |  |  |  |
| 9 | Observe for signs of rebleeding for 4 hours |  |  |  |  |
| 10 | Discontinue the gastric aspiration port from intermittent suction |  |  |  |  |
| 11 | Using a syringe, aspirate any remaining air from the esophageal and gastric balloons |  |  |  |  |
| 12 | Gently remove the Blakemore tube |  |  |  |  |
