# Supplementary material for: Sengstaken–Blakemore Tube Placement: A Simulation-Based Training Program for a High-Acuity, Low-Frequency Procedure
Source: MedEdPORTAL. 2026 Jun 24;22:11613. doi: 10.15766/mep_2374-8265.11613 (PMC13291162; doi:10.15766/mep_2374-8265.11613)
Supplement: Supplementary file 1 — Components of SBT Kit.docxSimulation Case.docxBlakemore Tube Placement Checklist.docxBlakemore Placement Pretraining Survey.docxBlakemore Placement Posttraining Survey.docx [file mep_2374-8265.11613-s001.zip › D. Blakemore Placement Pretraining Survey.docx]

# Appendix D: Blakemore Placement (Pre-Training Survey)

Current level of training/department *

( ) GI fellow
( ) GI attending
( ) ICU (MICU/SICU; PA, resident, fellow, attending)

Have you received formal training on Blakemore tube placement prior to this initiative? *

( ) Yes
( ) No

How many times have you placed a Blakemore tube in the past? *

( ) 0
( ) 1-2
( ) 3-4
( ) 5 or more

When was the last time you placed a Blakemore tube (i.e., 6 months ago, 5 years ago)?

_____________________________________________________________

How confident are you with Blakemore tube placement? *

Very uncomfortable 1 2 3 4 5 Very comfortable

How confident are you with Blakemore tube management? *

Very uncomfortable 1 2 3 4 5 Very comfortable
