# Supplementary material for: Sengstaken–Blakemore Tube Placement: A Simulation-Based Training Program for a High-Acuity, Low-Frequency Procedure
Source: MedEdPORTAL. 2026 Jun 24;22:11613. doi: 10.15766/mep_2374-8265.11613 (PMC13291162; doi:10.15766/mep_2374-8265.11613)
Supplement: Supplementary file 1 — Components of SBT Kit.docxSimulation Case.docxBlakemore Tube Placement Checklist.docxBlakemore Placement Pretraining Survey.docxBlakemore Placement Posttraining Survey.docx [file mep_2374-8265.11613-s001.zip › E. Blakemore Placement Posttraining Survey.docx]

# Appendix E: Blakemore Placement (Post-Training Survey)

Current level of training/department *

( ) GI fellow
( ) GI attending
( ) ICU (MICU/SICU; PA, resident, fellow, attending)

How confident were you with Blakemore tube placement BEFORE training? *

Very uncomfortable 1 2 3 4 5 Very comfortable

How confident are you with Blakemore tube placement AFTER training? *

Very uncomfortable 1 2 3 4 5 Very comfortable

How confident were you with Blakemore management BEFORE training? *

Very uncomfortable 1 2 3 4 5 Very comfortable

How confident are you with Blakemore management AFTER training? *

Very uncomfortable 1 2 3 4 5 Very comfortable

How helpful was the video tutorial in learning/facilitating Blakemore placement? *

Very unhelpful 1 2 3 4 5 Very helpful

How helpful was the checklist/manual in learning/facilitating Blakemore placement? *

Very unhelpful 1 2 3 4 5 Very helpful

How helpful was the SIM session in learning/facilitating Blakemore placement? *

Very unhelpful 1 2 3 4 5 Very helpful

Please provide feedback for any components of the Blakemore course (instructional checklist/video, SIM session)

_____________________________________________________________
_____________________________________________________________
_____________________________________________________________
