# Supplementary material for: Sengstaken–Blakemore Tube Placement: A Simulation-Based Training Program for a High-Acuity, Low-Frequency Procedure
Source: MedEdPORTAL. 2026 Jun 24;22:11613. doi: 10.15766/mep_2374-8265.11613 (PMC13291162; doi:10.15766/mep_2374-8265.11613)
Supplement: Supplementary file 1 — Components of SBT Kit.docxSimulation Case.docxBlakemore Tube Placement Checklist.docxBlakemore Placement Pretraining Survey.docxBlakemore Placement Posttraining Survey.docx [file mep_2374-8265.11613-s001.zip › A. Components of SBT Kit.docx]

**Appendix A:** Components of the SBT Kit


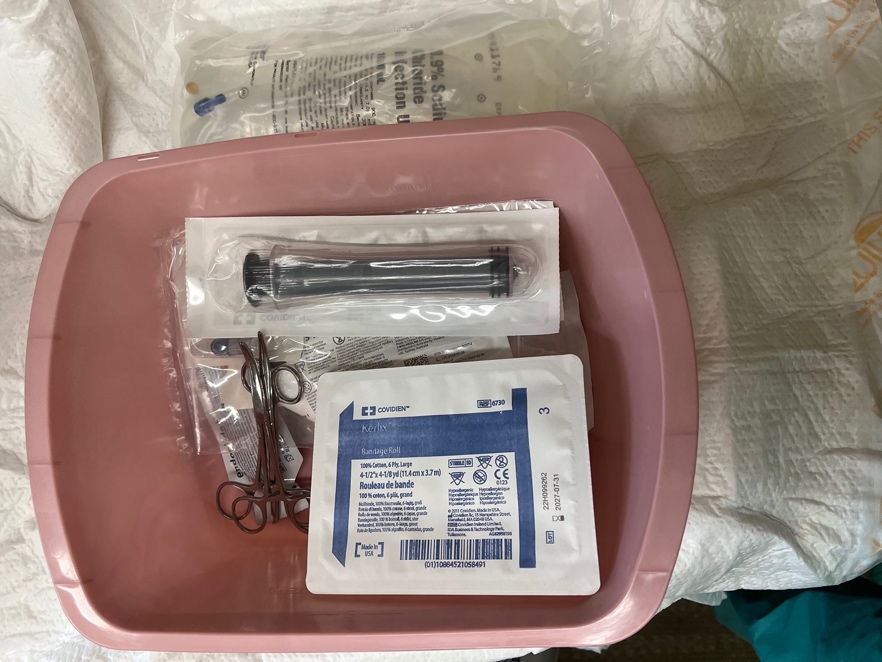


Author Owned.

(A) Washbasin (~7.4L)

(B) Locking scissor clamps (e.g., Kelly or hemostat clamp) (2)

(C) Syringes (50-60 cc tapered tip) (2)

(D) Syringes (50-60 cc luer lock) (2)

(E) Multipurpose tubing adapter (or a single-sided tapered Christmas tree with luer-lock end) (3)

(F) Double-sided Christmas tree adaptor (1)

(G) Blood pressure cuff (1)

(H) Dead end luer lock caps (2)

(I) Endotracheal (ET) tube holder (1)

(J) Forceps (3)

(K) Gauze bandage roll (1)

(L) Lubricating jelly (1)

(M) Scissors (1 pair)

(N) Fluid bags (1000 mL) or weight proxies (0.5-1 lb) (2)

(O) Three-way stopcocks (with two female luer lock ports and male luer slip connector, port covers, swivel) (3)

(P) Suction tubing (1)

(Q) Utility marker (1)
